# Genetic diversity of the submerged macrophyte *Ceratophyllum demersum* depends on habitat hydrology and habitat fragmentation

**DOI:** 10.3389/fpls.2023.1277916

**Published:** 2023-11-09

**Authors:** Attila I. Engloner, Kitti Németh, Péter B. Kós, Emese Meglécz, Judit Bereczki

**Affiliations:** ^1^ Institute of Aquatic Ecology, Centre for Ecological Research, Budapest, Hungary; ^2^ Institute of Plant Biology, Biological Research Centre, Eötvös Loránd Research Network, Szeged, Hungary; ^3^ Department of Biotechnology, Faculty of Science and Informatics, Szeged University, Szeged, Hungary; ^4^ Aix Marseille University, Avignon University, French National Center for Scientific Research (CNRS), French National Research Institute for Sustainable Development (IRD), Mediterranean Institute of Marine and Terrestrial Biodiversity and Ecology (IMBE), Marseille, France; ^5^ Molecular Taxonomy Laboratory, Hungarian Natural History Museum, Budapest, Hungary

**Keywords:** microsatellites, multilocus genotypes, vegetative dispersion, Danube, river channel, backwaters

## Abstract

The adaptability of plant populations to a changing environment depends on their genetic diversity, which in turn is influenced by the degree of sexual reproduction and gene flow from distant areas. Aquatic macrophytes can reproduce both sexually and asexually, and their reproductive fragments are spread in various ways (e.g. by water). Although these plants are obviously exposed to hydrological changes, the degree of vulnerability may depend on the types of their reproduction and distribution, as well as the hydrological differences of habitats. The aim of this study was to investigate the genetic diversity of the cosmopolitan macrophyte *Ceratophyllum demersum* in hydrologically different aquatic habitats, i.e. rivers and backwaters separated from the main river bed to a different extent. For this purpose, the first microsatellite primer set was developed for this species. Using 10 developed primer pairs, a high level of genetic variation was explored in *C. demersum* populations. Overall, more than 80% of the loci were found to be polymorphic, a total of 46 different multilocus genotypes and 18 private alleles were detected in the 63 individuals examined. The results demonstrated that microsatellite polymorphism in this species depends on habitat hydrology. The greatest genetic variability was revealed in populations of rivers, where flowing water provides constant longitudinal connections with distant habitats. The populations of the hydrologically isolated backwaters showed the lowest microsatellite polymorphism, while plants from an oxbow occasionally flooded by the main river had medium genetic diversity. The results highlight that in contrast to species that spread independently of water flow or among hydrologically isolated water bodies, macrophytes with exclusive or dominant hydrochory may be most severely affected by habitat fragmentation, for example due to climate change.

## 1 Introduction

Aquatic macrophytes are widely distributed and of great importance in various freshwater and marine ecosystems. They play a key role in aquatic food webs and biochemical cycles, influence the hydrology and sediment dynamics of aquatic habitats by altering current velocity, and provide habitat and refuge for many living organisms from microbiota to vertebrates and multiple benefits for humans (Bornette and Puijalon, 2011; Hossain et al., 2017; Thomaz, 2023). It is widely recognized that climate change threatens aquatic plants in several ways, including rising temperature, CO_2_ concentration and dissolved organic carbon, as well as changes in nutrient availability, light conditions and salinity. Climate change has been proven to greatly affect the growth and physiology of these plants, as well as the species composition and ecosystem functioning (Hossain et al., 2017; Li et al., 2017; Reitsema et al., 2018; Reitsema et al., 2020). First, aquatic macrophytes are completely dependent on the water supply of their habitat and are obviously exposed to hydrological changes caused by global climate change. It is clear that where less precipitation falls and the water supply of the habitats decreases, the living conditions of macrophytes deteriorate, or, if the climate becomes wetter, their potential habitats may increase. In river ecosystems, altered flow regimes have significant effects on macrophytes (Reitsema et al., 2020; Goldenberg-Vilar et al., 2022; Rivaes et al., 2022). Droughts and floods associated with climate change have both been shown to simplify physical habitats by leading to shallow flow conditions and reduced hydraulic diversity, especially in ecologically important pool and riffle features(O’Briain, 2019).

Not only the amount, presence or absence of water in a given habitat can affect the colonization of aquatic macrophytes, but also the degree of connectivity between different habitats can be of great importance. These plants can reproduce both sexually and asexually, and their seeds and vegetative shoot fragments can spread in several ways; by wind, birds or water (Santamaría, 2002; Capers, 2003; Jones et al., 2020). It is easy to understand, that dispersion by water flow (hydrochory) is not possible between hydrologically isolated habitats. In addition to sexual reproduction, gene inflow from distant areas can significantly increase the genetic diversity of populations, which can improve their ability to adapt to changing environments (Cao et al., 2020; Cao et al., 2021). Conversely, if a plant species reproduces mainly vegetatively and has only limited ‘external’ propagating material introduced into its population (or none at all), it is expected to be more vulnerable to habitat changes.

Changes in the genetic diversity of populations can be caused or enhanced by various environmental factors, among which habitat heterogeneity and dispersal can play decisive role (Orsini et al., 2013; Davis et al., 2018). The environmental factors have also been suggested as important drivers of genetic variation in aquatic plants, however, only a few studies focus on these organisms (Foust et al., 2016; Robertson et al., 2017; Li et al., 2022 and references therein). Temperature, salinity-gradient, heavy metals and bird-mediated dispersal have been shown to affect macrophyte genetic diversity (Gupta and Sarin, 2009; Triest et al., 2010; Cao et al., 2017; Li et al., 2022).

*Ceratophyllum demersum* is a cosmopolitan submersed macrophyte with wide climatic tolerance and all the ecosystem functions detailed above (Les, 1986; Qadri et al., 2022). This species is known for low sexual reproduction and predominance of clonal growth (Les, 1988; Les, 1991). Although the main reproductive mechanism of *C. demersum* is reported to be shoot fragmentation (Arber, 1920; Les, 1991; Fukuhara et al., 1997), and dispersion of this species relies on these vegetative fragments flowing in water rather than pollen or seeds (Wade, 1993; Capers, 2003), the effect of hydrological conditions on its genetic variability has never been studied.

Most of the molecular studies on *C. demersum* are phylogenetic (Moore et al., 2007; Szalontai et al., 2018; Albert and Renner, 2020; Yang et al., 2020), and further investigations concern the genetic alteration in response to heavy metal tolerance (Gupta and Sarin, 2009; Khaleel et al., 2022) and the isolation of genes encoding enzymes potentially required for heavy metal accumulation (Shukla et al., 2012). A variety of molecular markers and techniques have been used for the genetic investigations of *Ceratophyllum*, from karyology to AFLP, MSAP and ISSR markers (Triest et al., 2010; Cao et al., 2017; Gargiulo et al., 2022; Li et al., 2022), however, microsatellite primers and studies based on them have not yet been published for this species. Microsatellites (i.e. short tandem repeats) have been the most widely used markers for genotyping plants over the past 20 years (Vieira et al., 2016), as they are multi-allelic markers with a mutation rate of 10^3^-10^6^ per cell generation, which can be 10 orders of magnitude greater than point mutations (Gemayel et al., 2012). Their codominant nature, biparental mode of inheritance and elevated levels of polymorphism (Goldstein and Schlötterer, 1999; Schlötterer, 2000; Ellegren, 2004) have made them particularly informative and powerful for investigating genetic diversity and structure as well as demographic processes (Kim and Sappington, 2013). The advantages of using microsatellite markers, especially in population genetic studies, were even demonstrated for macrophyte species (Ouborg et al., 1999; Triest et al., 2010; Kong et al., 2019).

For the above reasons, the aim of the present paper is to explore whether the microsatellite polymorphism of the worldwide macrophyte *C. demersum* depends on the hydrology of the habitat and how it can affect the genetic diversity of its populations to changing environments. For this purpose, we have developed and provide the first microsatellite primers set for this species.

## 2 Materials and methods

### 2.1 Marker development

To produce high-quality DNA extract, *C. demersum* samples were first rinsed and impurities were removed with toothbrushes. The stems were then discarded and the leaves were used for subsequent processes. About 80 g leaf material were grinded in 200 ml of “grinding buffer” (450 mM Sucrose, 1.5 mM EGTA, 0.2% [w/v] bovine serum albumin [BSA], 0.6% [w/v] polyvinylpyrrolidone 40, 10 mM dithiothreitol [DTT], 0.2 mM phenylmethylsulfonyl fluoride [PMSF], and 15 mM MOPS [3-(N-morpholino)-propanesulfonic acid]/KOH, pH 7.4). The cells were disrupted by homogenizing for three periods of 15 s using a Waring blender. Cell debris was removed by centrifugation at 3,000 g for 5 min. According to the recommendations, the sample DNA was isolated using Thermo Scientific™ Genomic DNA Purification Kit (Thermo Fisher Scientific, Waltham, MA, USA) and sequenced on Illumina Miseq sequencer using v3 kit for 2x300 bp PE protocol. The sequences were aligned using Mira4 (Chevreux et al., 1999) in accurate mode and the contigs exceeding 1 kb length were used for microsatellite prediction.

The potential SSR loci were mined from the assembled, total of 289 contigs, by QDD v. 3.1.2 (Meglécz et al., 2014) using default parameters. The following criteria were used to select primer pairs for laboratory testing: (i) the sequence contained only pure microsatellites in the target region with at least six repeats; (ii) did not contain repeats of (AT)n or (CG)n; (iii) the primer alignment score to the amplified sequence was lower than six; (iv) primers were at least five bases away from the microsatellite motif; (v) no BLAST hit to non-Viridiplantae sequences in GenBank; and only one microsatellite was selected from each contig to avoid linkage disequilibrium. After the above selection, a total of 50 primer pairs were selected for initial screening.

Among the potential primers tested according to the protocol detailed in the next section, 16 consistently amplified the target sequences and showed signs of variability between the tested individuals based on visual inspection of the agarose gel. Of the 16 loci, those with at least two alleles were retained for the final marker set.

Finally, our marker development resulted in 10 recommendable primer pairs to explore genetic polymorphism of *C. demersum* (**Table 1**). The use of these primers is facilitated by the same optimal annealing temperature and a wide range of the amplicon size, which allows multiplexing of PCR products in a single reaction.

**Table 1:** Characteristics of 10 microsatellite loci of *Ceratophyllum demersum*.

Locus	Primer sequence (5’-3’)		Repeat motif	Fluorescent dye label	T_a_ (C°)	Size range (bp)	N_a_	H_o_
HME4	fw	TTTAAATCCTTCTCCCATTCTTCAAA	ATC	NED	55	152-176	9	0.779
	rv	GCAGAACACATCTCTATCAATGGA						
HME6	fw	TTATGCCCAAGCTTATAATAGACTTGA	ATC	VIC	55	290-308	3	0.115
	rv	TCTTTCAAGAAGTGGATTTATGATTGT						
HME11	fw	GAGGAAGAGATTGGCCCTCC	AG	VIC	55	181-195	8	0.817
	rv	TCCATGATGATCGCTGACCA						
HME14	fw	GGTTGTCACCCTTCTTCTCTT	AC	FAM	55	94-106	4	0.316
	rv	CACAACCAGTGCGCGAATG						
HME17	fw	TCTGGAAGAGAAGAACAAAGTTTCA	AGAT	FAM	55	124-144	6	0.562
	rv	GCCTGACAATCATACATGTATAGACC						
HME24	fw	CCACTGATAGTAATTGCCTCCT	ATC	VIC	55	119-125	2	0.013
	rv	AGAATTCATGTAGCCGACCC						
HME27	fw	TCTTGTTAGGCCTTATTCATAGTTTC	AG	PET	55	160-226	11	0.756
	rv	TGACTAAACGCTGCTCTGGA						
HME29	fw	ACCTACCTGCTGTATTTGGCT	AC	PET	55	298-304	4	0.444
	rv	ACAGCAATCGAACTGTTCTGC						
HME32	fw	TCCCTATTGGACTGCATATTTCAT	AG	NED	55	189-205	6	0.967
	rv	GTGCAGGCTTCTTCTCTTGG						
HME43	fw	GGATTTAGGTTGTACTTTGCGC	AAG	NED	55	82-88	3	1.000
	rv	ACTCGGACAACGGAAACAGT						

Values are based on the analyses of 63 individuals. T_a_, optimal annealing temperature; N_a_, number of alleles per locus; H_o_, observed heterozigosity.

### 2.2 Sampling and laboratory work to explore microsatellite polymorphism of Ceratophyllum populations

**Figure 1:**
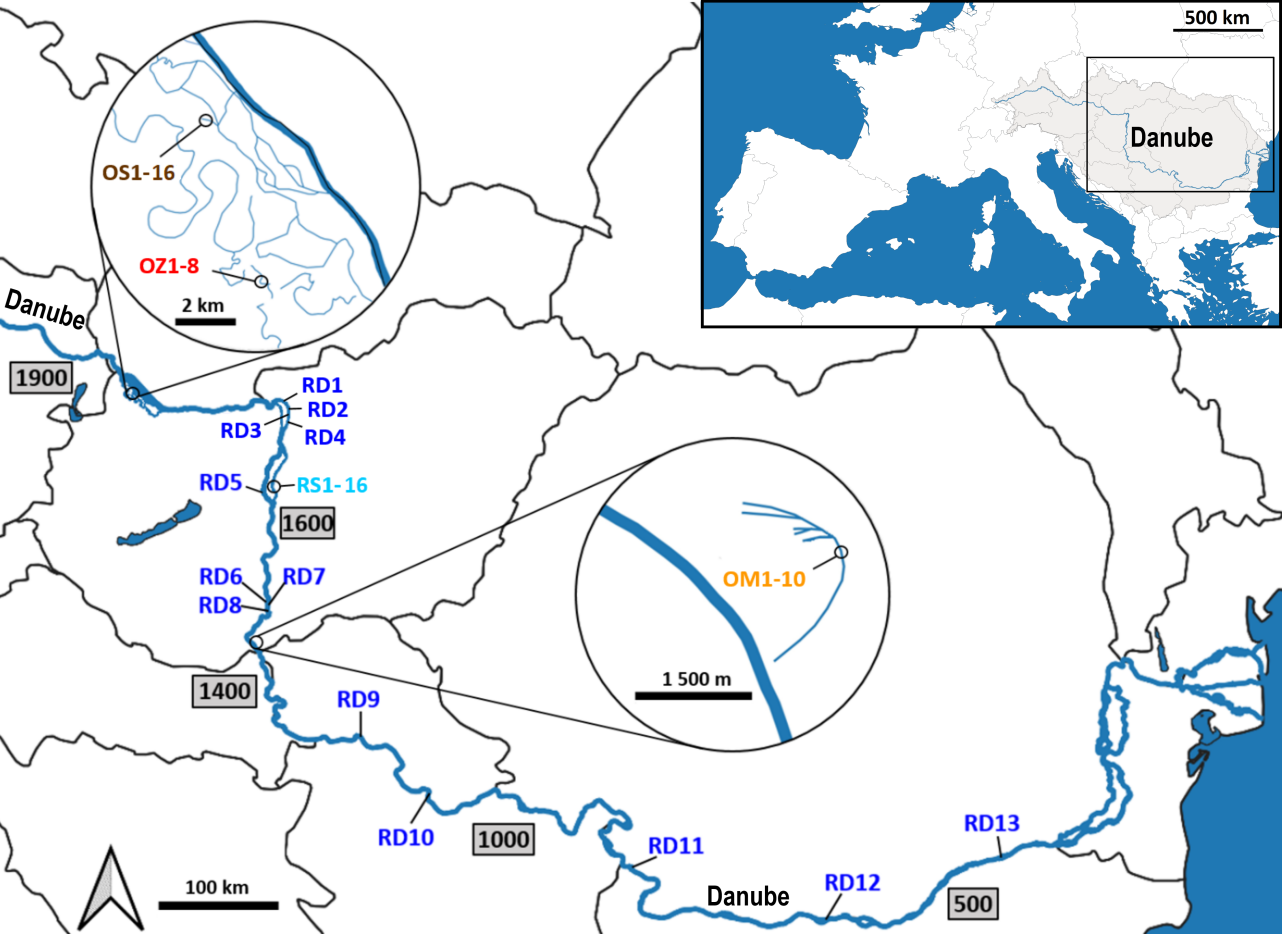
Sampling locations along the Danube River. RD – the main Danube channel; RS, Soroksári Danube; OS, Schisler oxbow; OM, Mocskos Danube; OZ, Zátonyi Danube. The numbers after the abbreviations indicate the serial numbers of samples, while numbers in the rectangles show the river kilometers in the main river channel.

Plant material was collected from five aquatic habitats belonging to two main different hydrological types (**Figure 1**). Two habitats represent rivers (hereinafter referred to as ‘river habitats’), namely (i) the main channel of the River Danube (RD) and (ii) the Soroksári Danube (RS), the second largest side arm of River Danube in Hungary between the 1642 and 1586 river kms. The latter is closed at both ends by sluices regulating its water flow. Due to the hydrological regulation, only a small amount of water enters the side arm from the main channel, it is the fortieth to sixtieth part of the Danube’s water at mean water-flow (Hungarian Hydrological Forecasting Service, 2023). In addition to the two ‘river habitats’, three oxbows were also selected; (iii) Schisler oxbow (OS) and (iv) Zátonyi Danube (OZ) in the Szigetköz, the watery plain with many branches, islands and backwaters between the main Danube channel and the Moson-arm, and (v) the oxbow Mocskos Danube (OM), a former Danube side branch lying on the floodplain of Béda-Karapancsa Landscape Protection Area. Of the five habitats, only the main Danube channel and the Soroksári Danube have water flowing continuously, and only these habitats have steady longitudinal connections with distant river sections. Among the backwaters, the Mocskos Danube can temporarily connect to the Danube during the river’s floods, however, these events have only occurred on a third of the days in the last fifty years (Hungarian Hydrological Forecasting Service, 2023).

The two selected ‘river habitats’ allowed both large-scale and fine-scale sampling: 13 plants were collected between the 1680-425 river kms of the main Danube channel, and 16 samples were taken along the 1600 m long stretch of the Soroksári Danube, at river km 31. Sampling in the oxbows was determined by the size of the water bodies and the amount of plant cover in them. Furthermore, in order to avoid sampling from the same plant (i.e. collecting multiple leaves of the same individual), a distance of at least 30 m was kept between two samples. In this way, the *C. demersum* stands in the 500, 400 and 900 meter long sections of OS, OZ and OM provided 16, 8 and 10 plant samples for the genetic investigations. Plant material was stored at 4°C during transport to the laboratory, and then at -80°C until DNA isolation.

DNA was extracted by homogenizing 200 mg frozen leaves in 800 μl CTAB isolation buffer (2%) following the protocol described in Bousquet et al. (1990). The amplification procedure from 5 μl of DNA extracts was carried out in 15 μl final reaction volumes containing 10X PCR buffer, 3 mM MgCl_2_, 0.2 mM dNTPs, 0.05 units/μl of Taq DNA polymerase (Taq DNA Polymerase, recombinant, Fermentas) and 0.5 μM of each primer. Forward primers were labelled fluorescently at their 5’ end. The following cycling conditions were used: initial denaturation for 5 min at 94°C, 40 cycles of 45 s at 94°C, 45 s at 55°C, 45 s at 72°C; final elongation of 10 min at 72°C. The success of PCR was checked by running 2 µl of product on 1.4% agarose gels stained with GelRed Nucleic Acid Stain (Biotium Inc., Fremont, CA, USA). After amplification, PCR products were multiplexed in a single reaction and fragment analysis was carried out on an ABI 3130 Genetic Analyser. Allele sizes were estimated using Peak Scanner software (Thermo Fisher Scientific, Waltham, MA, USA).

### 2.3 Statistical analyses

Checking for the presence of null alleles was performed using Micro-Checker 2.2.3 (Van Oosterhout et al., 2004) by Monte Carlo simulation of expected homozygote frequencies and heterozygote allele size differences. Since null alleles were only observed in a single case, the entire dataset was used for further analyses. Parameters of polymorphism were determined using GenAlEx v. 6.5 (Peakall and Smouse, 2006; Peakall and Smouse, 2012) and Fstat v. 2.9.4 (Goudet, 2003). Based on the genetic distance matrix calculated by Identix v.1.1 (Belkhir et al., 2002), UPGMA tree was constructed using PAST v. 4.12. A minimum spanning network was generated by the bruvo.msn() function of the ‘poppr’ package v. 2.9.3 in R (Kamvar et al., 2014; Kamvar et al., 2015) using the Bruvo method (Bruvo et al., 2004) to calculate genetic distances. To analyse the correlation between the genetic and geographic distance matrices, a Mantel test was carried out using GenAlEx v. 6.5 (Peakall and Smouse, 2006; Peakall and Smouse, 2012) with 9999 permutations. Genetic distances among individuals were calculated by GenAlEx v. 6.5, while the geographic distance matrix was produced by PAST v. 4.12 (Hammer et al., 2001).

The genetic structure of the populations has been analysed using Bayesian-clustering method (Pritchard et al., 2000). The most probable number of genetically differentiated groups (K) in the populations was estimated and the individuals were assigned to these groups. Structure 2.3.4 was run to carry out these analyses using default settings with an initial burn in of 100,000 steps and running length of 500,000 steps. In the evaluation of the results, ΔK was computed which indicates the change in log probability between successive K values (Evanno et al., 2005). Structure Harvester Web 0.6.94 (Earl and vonHoldt, 2012) was used to compute the ΔK values. The package ‘pophelper’ in R (Francis, 2017) was applied to average the ten runs of the most probable K value given by Structure and correct for label switching.

To reveal the genetic differentiation among plant samples from the five selected habitats, the microsatellite allele frequency data were evaluated by standardized principal component analysis (PCA) using SYN-TAX 2000 computer program package (Podani, 2001).

## 3 Results

### 3.1 Level of microsatellite polymorphism

All indices of polymorphism indicated a high level of variation in *C. demersum* populations (**Table 2**). Overall, more than 80% of the loci were found to be polymorphic. The average number of alleles per locus was slightly more than 3 and the average frequency of heterozygotes was over 57%. A total of 46 different multilocus genotypes and 18 private alleles were detected in the 63 individuals examined. The variability parameters corrected for sample size, i.e. the number of effective alleles and the allelic richness in total were 2.222 and 2.905, respectively.

**Table 2:** The parameters of variability based on 10 microsatellite loci.

Habitat	N	N_a_	N_e_	AR	N_PA_	MG	I	P%	H_o_	H_e_
**RD**	13.00	4.600	2.744	3.996	9	12	1.039	90.0	0.538	0.517
**RS**	15.80	3.700	2.390	3.216	3	10	0.867	90.0	0.560	0.457
***River total***	*14.40*	*4.150*	*2.567*	*3.606*	*6*	*11*	*0.953*	*90.0*	*0.549*	*0.487*
**OS**	15.80	2.400	2.179	2.377	0	13	0.703	70.0	0.663	0.425
**OZ**	8.00	2.300	1.785	2.300	1	3	0.567	70.0	0.613	0.355
**OM**	10.00	2.700	2.012	2.635	5	8	0.724	90.0	0.510	0.425
***Oxbow total***	*11.27*	*2.467*	*1.992*	*2.437*	*2*	*8*	*0.665*	*76.7*	*0.595*	*0.402*
**Total**	12.52	3.140	2.222	2.905	18	46	0.780	82,0	0.577	0.436

N, sample size; N_a_, average number of alleles per locus; N_e_, the number of effective alleles; AR, allelic richness (N=8); N_PA_, number of private alleles; MG, the number of multilocus genotypes; I, Shannon’s information index; P%, percentage of polymorphic loci on the basis of the 95% criterion; H_o_, observed heterozygosity; H_e_, expected heterozygosity. RD, the main Danube channel; RS, Soroksári Danube; OS, Schisler oxbow; OM, Mocskos Danube; OZ, Zátonyi Danube.

The data clearly show greater genetic variability in river populations than in backwaters (e.g., N_e_ 2.567 and 1.992; AR 3.606 and 2.437, for the former and the latter, respectively). The average number of alleles per locus, the number of effective alleles, allelic richness and Shannon’s information index all reached the highest values in the Danube River and the Soroksári side arm, while among the backwaters, most of these values were the highest in the Mocskos oxbow (**Table 2**).

### 3.2 Structure of the genetic variation

**Figure 2:**
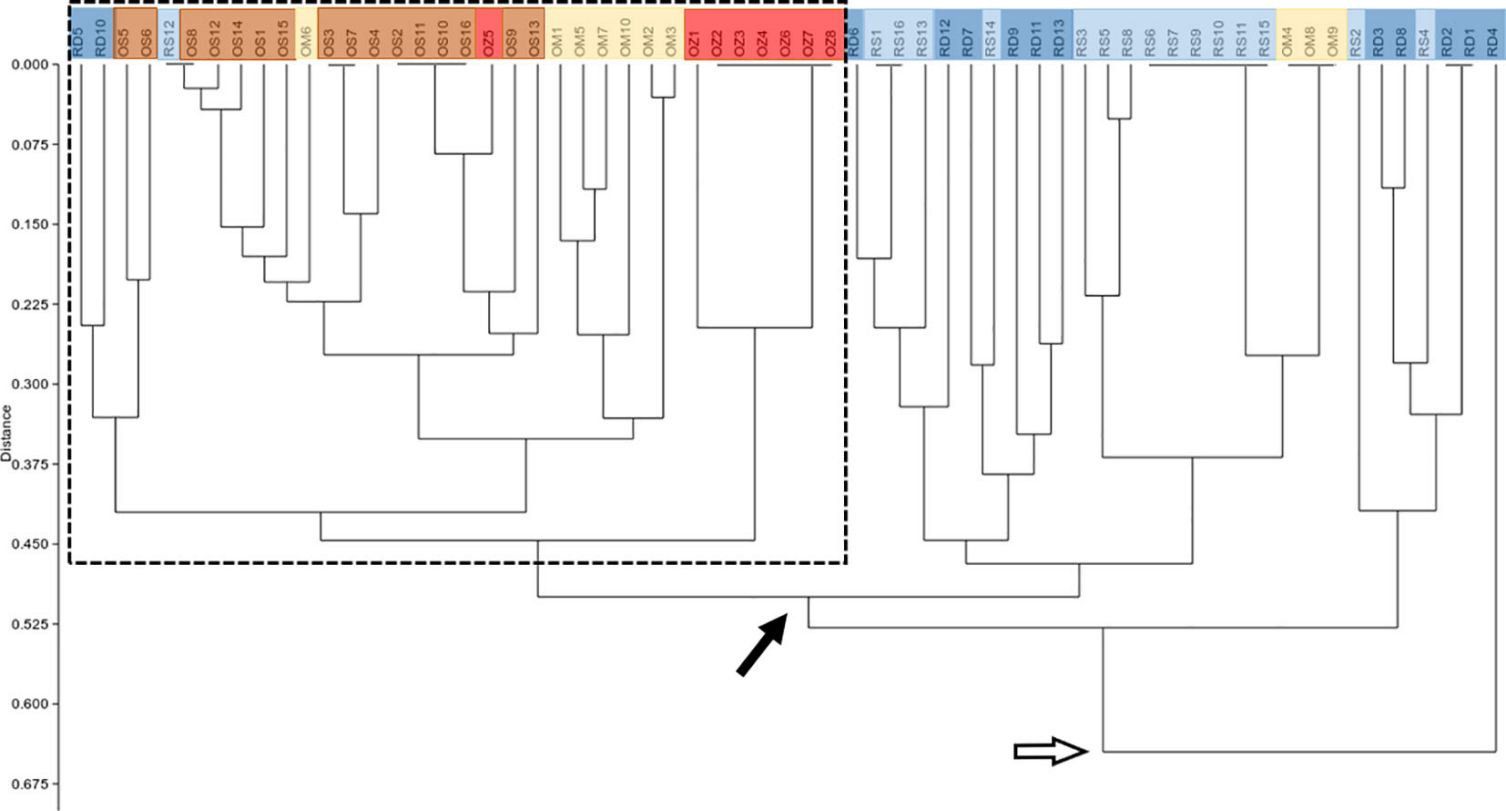
UPGMA tree based on genetic distance matrix among individuals. RD, the main Danube channel; RS, Soroksári Danube; OS, Schisler oxbow; OM, Mocskos Danube; OZ, Zátonyi Danube. Samples from river habitats and oxbows are indicated by bluish and yellowish-red colors, respectively. The dashed box shows the main clade that contains the majority of oxbow plants. Black and empty arrows indicate clades that contain all backwater and river samples, respectively.

**Figure 3:**
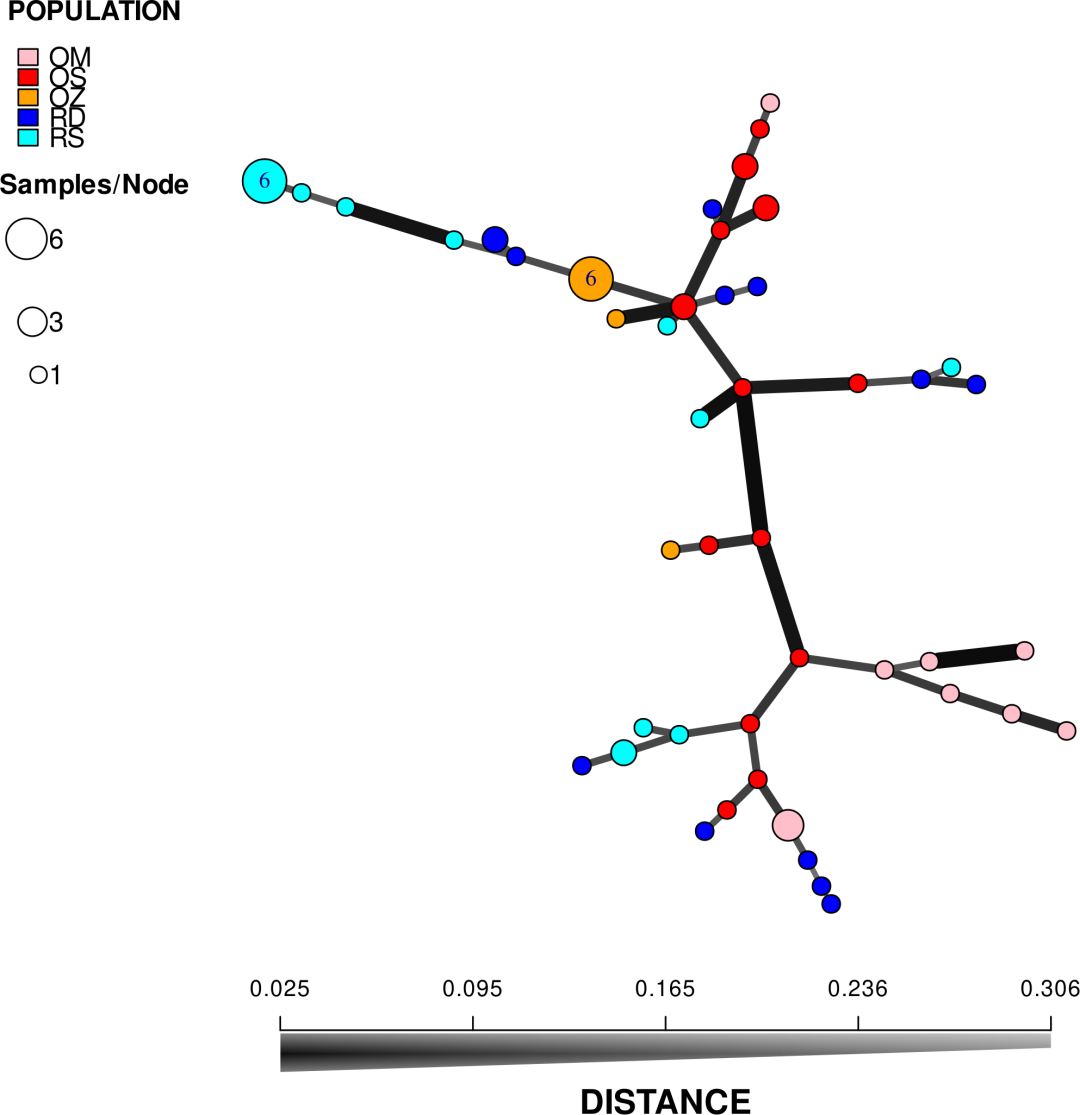
Minimum spanning network based on Bruvo’s distance. Each node on the graph represents a different multilocus genotype, while the edges show the genetic distances between them. Areas of circles are proportional to the number of multilocus genotypes displayed. RD, the main Danube channel; RS, Soroksári Danube; OS, Schisler oxbow; OM, Mocskos Danube; OZ, Zátonyi Danube.

According to the hierarchical classification, none of the populations formed completely separate clusters, i.e. samples from the different habitats have mixed with each other to some extent (**Figure 2**). However, with the exception of three samples of Mocskos Danube oxbow, plants of backwater habitats were included in one main clade (marked with a dashed box at the hierarchical level of 0.450 on the UPGMA tree). The largest distance between all oxbow samples was 0.500 (marked with black arrow in **Figure 2**). Contrary, plants from the two river habitats showed higher mixing and dissimilarity (the latter indicated by an empty arrow at the distance level of 0.650 on the UPGMA tree). Geographically closer samples were not clearly placed in closer clades, which is consistent with the result of Mantel test showing a statistically significant positive but weak correlation between geographic and genetic distance (R=0.249, p=0.0001). It can also be observed in **Figure 2**, that multilocus genotypes consisting of several plant samples occurred in both river habitats and backwaters (up to six plants per genotype, in both habitat types). Minimum spanning network also shows that plants belonging to the river habitats can appear quite far from each other, and larger circles, i.e. multilocus genotypes with more than one sample, are colored differently, indicating that they were observed in all habitats (**Figure 3**).

**Figure 4:**
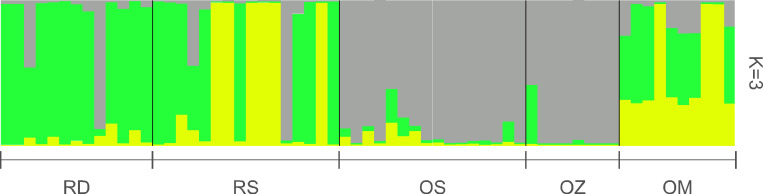
Bayesian assignment of individuals based on 10 microsatellite loci. RD, the main Danube channel; RS, Soroksári Danube; OS, Schisler oxbow; OM, Mocskos Danube; OZ, Zátonyi Danube.

According to the Structure analysis, the most probable number of genetically differentiated groups (K) proved to be three (**Figure 4**). The plants of the main Danube channel consisted predominantly of a single genetic cluster, in addition to which, another genetic cluster appeared in high proportion in the Soroksári Danube. The samples of these two river habitats were clearly separated from those of Schisler and Zátonyi oxbows, which were mainly characterized by the third cluster. In the Mocskos Danube, all three clusters were mixed.

**Figure 5:**
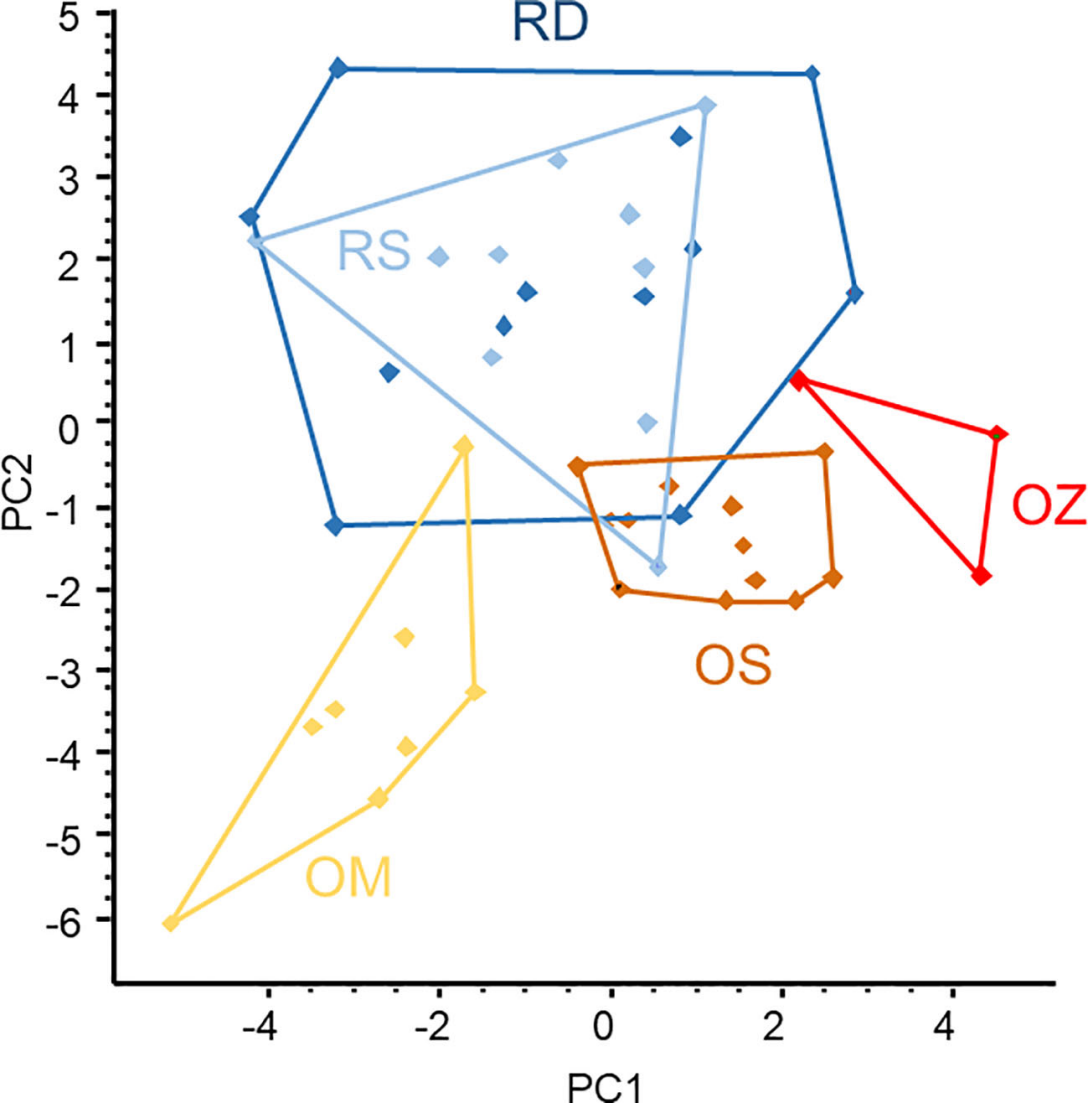
PCA ordination of *Ceratophyllum demersum* samples based on microsatellite allele frequency data. Convex polygons enclose plant samples collected from the same habitats. RD, the main Danube channel; RS, Soroksári Danube; OS, Schisler oxbow; OM, Mocskos Danube; OZ, Zátonyi Danube.

The ordination of microsatellite data resulted in two large, overlapped groups of samples taken from the main Danube River and the Soroksári side arm, and three smaller and better-separated groups representing the oxbow samples (**Figure 5**). This means that the populations of the three oxbows were separated from each other while the plants of two river habitats were mixed based on microsatellites. As the areas of the polygons enclosing objects in the scattergram are proportional to the variability between the objects (i.e. the larger the area the higher the variability), the result demonstrated that genetic variability of *C. demersum* in river habitats was higher than in backwaters. Comparing the backwater samples, the plants of the Mocskos oxbow showed the highest variability.

## 4 Discussion

Although microsatellites have become the most widely used markers for genotyping plants, and the benefits of their use were demonstrated even in population genetic studies of macrophytes, microsatellite primers have not yet been published for *C. demersum*. Based on the newly developed loci, this study revealed differences in the microsatellite polymorphism of *C. demersum* populations related to the hydrological types of the habitats. Although the number of samples obtained from the populations was not exactly the same due to differences in size and vegetation cover of the water bodies, the number of effective alleles and allelic richness make it possible to compare the genetic variability of populations with different sample sizes. Both calculated variability parameters showed greater genetic variability in riverine habitats than in backwaters. In addition, the applied multivariate analyses revealed low genetic variability from backwaters based on both the smaller (Zátonyi and Mocskos oxbows) and larger sample sizes (Schisler oxbow). The results also demonstrated that a high number of multilocus genotypes could be detected from both river and backwater populations, however, the observed genotypes were genetically closer to each other in the latter than in the former habitat.

Genetic variation is highly dependent on the ratio of sexual and asexual reproduction, as well as the short- and long-distance dispersal of sexual and vegetative propagules (Eckert et al., 2016). Asexual spread is often assumed to be important in local, short-distance dispersal while regional, long-distance dispersal is thought to be based on sexual reproduction (Santamaría, 2002). However, the low sexual reproduction and the predominance of clonal growth and vegetative dispersion of *C. demersum* (Les, 1988; Les, 1991; Wade, 1993; Capers, 2003) suggest that the revealed genetic variability is the result of differences in vegetative propagation. In addition, the clear relationship observed between the genetic diversity of populations and the hydrological types of the habitats indicates that the mode of propagule spread is mainly hydrochory. The greatest genetic variability was revealed in river populations, where continuously flowing water provides a constant longitudinal connection with distant habitats. Contrary, *C. demersum* stands in the hydrologically isolated backwaters showed the lowest genetic diversity. The population of Mocskos oxbow, occasionally flooded by the Danube, which enables gene flow, had medium genetic diversity. If the majority of *C. demersum* propagules were not spread by water but by wind or birds, they could easily reach the backwaters that are hydrologically isolated from the main river but geographically very close to it.

Topography and geographic distance affect population genetic structure of plants, especially in aquatic habitats (Kong et al., 2019). However, unlike ponds and isolated backwaters that are discrete in terrestrial landscapes, water flows continuously in rivers and interconnected aquatic habitats, and the longitudinal connection of distant sections is ensured in these habitats. The importance of water flow in the establishment of plants was also demonstrated in the case of other submerged macrophytes, for which, however, seeds and the wind and animal-mediated dispersal were of great importance (Chen et al., 2009; Pollux et al., 2009; Triest et al., 2010). The spread of these macrophytes can be significant even against the water flow (Chen et al., 2009; Jones et al., 2020) or between water bodies with no hydrological connection, potentially providing their populations with high genetic diversity. Conversely, when hydrochory is exclusive or dominant, hydrological connections between habitats are crucial for gene flow, as the results of the present study suggest for *C. demersum*.

Of course, genetic diversity detected at marker loci in clonally spreading populations may be attributed to somatic mutations, which are enhanced by rapid clonal growth and a large amount of vegetative propagules (de Witte and Stöcklin, 2010; Eckert et al., 2016; Zhu et al., 2017; Kong et al., 2019). However, there are no reports on somatic mutation related to *C. demersum*.

For the above reasons, although habitat deterioration affects all macrophytes, the loss of hydrological connections between habitats is expected to be more severe for species, such as *C. demersum*, whose population genetic diversity is highly dependent on flow conditions and hydrological connectivity. The harmful effects of this kind of habitat fragmentation may appear as a result of any local anthropogenic intervention (cf. Cao et al., 2021) or global processes, such as climate change. The effect of the latter is also enhanced by temperature changes, which further reduce the genetic diversity of macrophytes by altering reproductive strategies, resulting in fewer flowers and thus less sexual reproduction (Li et al., 2017). That is, the main processes of climate change can reinforce each other, impairing the adaptability of macrophyte populations and endangering their ecosystem functions.

## Data availability statement

The sequence data has been deposited in the SRA database of NCBI with the code PRJNA1012867.

## Author contributions

AE: Conceptualization, Data curation, Formal Analysis, Funding acquisition, Investigation, Methodology, Visualization, Writing – original draft. KN: Investigation, Visualization, Writing – original draft. PK: Investigation, Methodology, Writing – original draft. EM: Investigation, Methodology, Writing – original draft. JB: Formal Analysis, Investigation, Methodology, Visualization, Writing – original draft.
